# Regional Absorption of Fimasartan in the Gastrointestinal Tract by an Improved In Situ Absorption Method in Rats

**DOI:** 10.3390/pharmaceutics10040174

**Published:** 2018-10-03

**Authors:** Tae Hwan Kim, Soo Heui Paik, Yong Ha Chi, Jürgen B. Bulitta, Da Young Lee, Jun Young Lim, Seung Eun Chung, Chang Ho Song, Hyeon Myeong Jeong, Soyoung Shin, Beom Soo Shin

**Affiliations:** 1 College of Pharmacy, Catholic University of Daegu, Gyeongsan, Gyeongbuk 38430, Korea; thkim@cu.ac.kr; 2 College of Pharmacy, Sunchon National University, Sunchon, Jeonnam 57992, Korea; shwhite@sunchon.ac.kr; 3 Central Research Institute, Boryung Pharm. Co., Ltd., Seoul 03127, Korea; yongha.chi@gmail.com; 4 College of Pharmacy, University of Florida, Orlando, FL 32827, USA; JBulitta@cop.ufl.edu; 5 School of Pharmacy, Sungkyunkwan University, Suwon, Gyeonggi-do 16419, Korea; dayoung717@skku.edu (D.Y.L.); panacea89@skku.edu (J.Y.L.); jsehome08@skku.edu (S.E.C.); sky84312@skku.edu (C.H.S.); wise219143@skku.edu (H.M.J.); 6 College of Pharmacy, Wonkwang University, Iksan, Jeonbuk 54538, Korea; shins@wku.ac.kr

**Keywords:** regional absorption, intestinal permeability, in situ single-pass perfusion, fimasartan, controlled release formulations

## Abstract

The aim of the present study was to assess the regional absorption of fimasartan by an improved in situ absorption method in comparison with the conventional in situ single-pass perfusion method in rats. After each gastrointestinal segment of interest was identified, fimasartan was injected into the starting point of each segment and the unabsorbed fimasartan was discharged from the end point of the segment. Blood samples were collected from the jugular vein to evaluate the systemic absorption of the drug. The relative fraction absorbed (*F_abs,relative_*) values in the specific gastrointestinal region calculated based on the area under the curve (AUC) values obtained after the injection of fimasartan into the gastrointestinal segment were 8.2% ± 3.2%, 23.0% ± 12.1%, 49.7% ± 11.5%, and 19.1% ± 11.9% for the stomach, duodenum, small intestine, and large intestine, respectively, which were comparable with those determined by the conventional in situ single-pass perfusion. By applying the fraction of the dose available at each gastrointestinal segment following the oral administration, the actual fraction absorbed (*F′_abs_*) values at each gastrointestinal segment were estimated at 10.9% for the stomach, 27.1% for the duodenum, 40.7% for the small intestine, and 5.4% for the large intestine, which added up to the gastrointestinal bioavailability (*F_X_·F_G_*) of 84.1%. The present method holds great promise to assess the regional absorption of a drug and aid to design new drug formulations.

## 1. Introduction

Following oral administration, a drug must pass through the gastrointestinal lumen, penetrate through the gut wall, and resist metabolic degradation by intestinal and hepatic enzymes, and biliary excretion [1]. In this process, the oral drug absorption is dependent on various factors including the pH, solubility and dissolution of a drug in the intestinal fluid, permeability across the intestinal membrane, presystemic metabolism, and drug transporters. Moreover, these factors vary depending on the location of the gastrointestinal tract [2]. Due to the interplays of these regional differences in the gastrointestinal environment and the physicochemical properties of a drug, the orally administered drug may have a favorable region for absorption. A better understanding of the dynamic and variable absorption process is essential for the successful development of oral dosage formulations. It helps to rationally design drug formulations with optimized bioavailability. Moreover, information regarding the regional differences of the gastrointestinal physiology and the factor-controlling absorption is especially critical to design controlled-release formulations with specific drug release pattern in the gastrointestinal tract.

Fimasartan is the 9th angiotensin II receptor antagonist approved for the treatment of mild to moderate hypertension with the brand name of Kanarb^®^. As a pyrimidine-4(3*H*)-one derivative of losartan, fimasartan provides greater potency and efficacy than losartan in parallel with the rapid onset of antihypertensive effects [3,4,5]. Following oral administration, fimasartan is known to be rapidly absorbed with an oral bioavailability of 32.8–44.7% in rats (solution), 8.0–17.3% in dogs (solution), and 18.6% ± 7.2% in humans (tablet) [6,7,8]. More than 90% of circulating fimasartan moieties in the plasma is the parent form suggesting fimasartan is metabolically stable, and fecal elimination and biliary excretion are the predominant elimination pathways of fimasartan [6]. Fimasartan has been licensed out to various countries worldwide including 13 Latin American countries as well as Russia and China. Recently, fixed dose combination tablets of fimasartan with another class drug, such as hydrochlorothiazide, amlodipine, and rosuvastatin, have been launched, and various preclinical and clinical studies are also ongoing to develop new formulations of fimasartan. 

Several experimental models are currently available to determine the intestinal absorption of a drug and the controlling mechanisms of absorption [9]. For example, immobilized artificial membrane (IAM) chromatography [10] and parallel artificial membrane permeability assay (PAMPA) [11] provide relatively simple and efficient screening tools to predict passive intestinal transport in the drug discovery stage. Various in vitro methods have been used to evaluate the intestinal absorption potential of drug candidates, which include animal tissue-based methods, such as everted gut techniques [12], Ussing chambers [13], and isolated membrane vesicles [14], and cell-based methods such as Caco-2 cells [15] and Madin-Darby canine kidney cells [16]. On the other hand, in vivo evaluation of drug absorption in animals is commonly used to predict the extent of absorption of drug candidates in humans. These experimental models have their own advantages and disadvantages, and the judicious use of the various techniques at the right stage of drug discovery and development is important. Furthermore, exciting and novel approaches have been extensively investigated to overcome the hurdles associated with poor gastrointestinal stability and absorption of biological drugs [17,18]. Accurate assessment of oral absorption by using proper experimental tools is also critical for the successful development of formulations and oral delivery strategies for biological drugs. 

Among the experimental models, in situ single-pass perfusion is a frequently used method to evaluate the regional intestinal permeability as well as the absorption kinetics of drugs [19,20,21]. In this method, the compound of interest is monitored in a perfusate and the difference between inlet and outlet concentrations, i.e., the loss of the compound, is attributed to the permeability. It has been suggested that the extent of absorption in humans can be predicted from single-pass intestinal perfusion studies in rats [21,22,23]. The major advantage of the single-pass perfusion method is the presence of intact blood and nerve supply in the experimental animals, which provides conditions close to the physiological state following oral administration [9]. The control of the factors, such as concentration, pH, and intestinal perfusion rate [20], is another strength of the in situ single-pass perfusion method. Moreover, it provides a unique ability to study regional differences in the gastrointestinal tract by using different gastrointestinal segments [24]. 

Nevertheless, the in situ single-pass perfusion method has limitations in that perfusion may disturb the normal physiology of the gastrointestinal tract and it does not consider other factors affecting drug concentrations in the intestinal lumen. It is assumed that the disappearance of the drug from the intestinal lumen is attributed to the intestinal permeability. However, the decrease of the drug concentration in the perfusate may not be entirely dependent on the absorption of the drug into the systemic circulation, but also on the drug metabolism in the gastrointestinal tract. Thus, the in situ single-pass perfusion method may overestimate the intestinal permeability and absorption of drugs undergoing intestinal metabolism. Moreover, a significant amount of drug may be necessary to conduct the in situ single-pass perfusion method, because continuous perfusion is needed until the steady state is reached. Thus, it may not be appropriate in the early stage of drug development for candidate screening purpose.

In the present study, an improved in situ absorption method has been developed to assess the regional absorption of fimasartan and compared the results with those obtained by the in situ single-pass perfusion method. The improved in situ absorption model evaluated the absorption by measuring the resulting drug plasma concentrations after an injection of a drug into a specific segment of the gastrointestinal tract, instead of measuring the disappearance of a drug in the perfusate during perfusion. Therefore, it allowed evaluation of net absorption in the different gastrointestinal segments in the more physiological condition, where drug absorption occurs sequentially as the drug solution passes through the gastrointestinal tract.

## 2. Materials and Methods

### 2.1. Chemicals and Reagents

Fimasartan and the internal standard (BR-A-563) were provided by Boryung Pharm. Co., Ltd. (Seoul, Korea). High performance liquid chromatography (HPLC) grade acetonitrile, methanol, and distilled water were products of Mallinckrodt Baker, Inc. (Phillipsburg, NJ, USA). Formic acid was obatined from Aldrich Chemicals (Milwaukee, WI, USA).

### 2.2. Animals

The animal studies were approved by the ethics committee for the treatment of laboratory animals at the Catholic University of Daegu (IACUC-2012-005). Male Sprague–Dawley rats, weighing 250–300 g, were housed in a temperature of 22–24 °C and a relative humidity of 50% ± 10% with a standard 12-h light/dark cycle. 

### 2.3. Determination of Hepatic First-Pass Metabolism and Gastrointestinal Bioavailability (F_X_·F_G_)

After anesthetized by an intraperitoneal injection of urethane (1 g/kg), the rats were cannulated with a polyethylene (PE) tubing (0.58 mm i.d., 0.96 mm o.d., Natsume, Tokyo, Japan) in the right jugular vein. For drug administration, the animals were also cannulated in the intended routes of administration. The femoral vein and the portal vein was cannulated for intravenous injection and portal vein injection, respectively. For portal venous injection, the portal vein was exposed by an abdominal incision and a PE tube (0.28 mm i.d., 0.61 mm o.d., Natsume, Tokyo, Japan) was inserted into the portal vein, and the wound was closed by applying epoxy glue (Krazy Glue, IL, USA). An abdominal incision was also made in rats receiving an intravenous injection to maintain the same experimental conditions. Fimasartan was dissolved in distilled water and injected at doses of 0.1 and 0.3 mg/kg into the femoral or portal vein. Blood samples were collected from the jugular vein before and at 2 min, 5 min, 10 min, 15 min, 30 min, 1 h, 2 h, 4 h, and 8 h after the fimasartan administration. Plasma samples were harvested by centrifugation of the blood samples at 1500× *g* for 10 min.

The plasma concentration of fimasartan vs. time data were analyzed by the noncompartmental method using the Phoenix^®^ WinNonlin^®^ software (Certara, L.P., Princeton, NJ, USA). Fractions of the administered dose that escaped the first-pass metabolism by the liver (*F_H_*) was calculated as follows:

(1) $$F_{H}=\frac{{AUC}_{portal\;vein}}{{AUC}_{\;iv}}\cdot \frac{D_{iv}}{D_{portal\;vein}}$$

where *AUC* represents the area under the plasma concentration vs. time curve with the time from zero to infinity while *D* is the dose; and the subscript refers to the route of administration.

The gastrointestinal bioavailability (*F_X_·F_G_*) was derived by:

(2) $$F_{X}\cdot F_{G}=\frac{F}{F_{H}}$$

where *F_X_* is the fraction absorbed and *F_G_* is the fraction of the dose that escapes the gut wall metabolism, and *F* is the absolute oral bioavailability of 39.85% [6].

### 2.4. In Situ Single-Pass Perfusion

After overnight fasting, the rats were anesthetized by an intraperitoneal injection of urethane (1 g/kg). For determination of the permeability in the duodenum, the abdomen was opened and the gastrointestinal segment of the duodenum (11 cm length from the end of the stomach) [25] was isolated and cannulated at both ends of the segment with a silicone tube (2 mm i.d. Daihan Scientific Co., Wonjoo, Korea). For determination of the permeability in the small intestine and large intestine, the same procedure was used to prepare the small intestine and large intestine segments. The segment of the small intestine (from the end of the duodenum to the caecum) and large intestine (from the caecum to the rectum) was isolated and cannulated. The cannulated segment was rinsed with 37 °C saline to clear the segment before perfusion. Each end of the cannulated segment was attached to the perfusion assembly, which consisted of a syringe pump (KD Scientific, Holliston, MA, USA) for input and a peristaltic pump (EP-1 Econo Pump, Bio-Rad, Hercules, CA, USA) for output. 

Fimasartan was dissolved in distilled water at a concentration of 0.1667 mg/mL and perfused with a perfusion rate of 0.2 mL/min. The animal was placed on a heating pad to maintain the body temperature, and the abdominal incision area was stapled to prevent loss of fluid and hypothermia. The outlet perfusates were collected on the ice at 10-min intervals from 50 to 140 min after the perfusion was initiated. The collected outlet samples were stored at −20 °C until analysis. The regional absorptive clearance (*P_e_A*) was estimated by:

(3) $$P_{e}A=Q_{in}\cdot ln(\frac{C_{in}}{C_{out}})$$

where *Q_in_* is the perfusion rate, and *C_in_* and *C_out_* are the inlet and outlet concentrations at the steady state, respectively. The regional fraction absorbed (*F_abs_*) at the segment *i* in the duodenum, small intestine, or large intestine was calculated as:

(4) $$F_{abs,\;i}=\frac{C_{in}-C_{out}}{C_{in}}$$

### 2.5. Improved In Situ Absorption Model

Similar to the in situ single-pass perfusion method, the rats were anesthetized by an intraperitoneal injection of urethane (1 g/kg) after overnight fasting. The abdomen was opened and the gastrointestinal segment of interest, i.e., stomach, duodenum, small intestine, or large intestine, was identified. The starting point of the segment was slightly tied off, the other end of the segment was cannulated with a silicone tube (2 mm i.d. Daihan Scientific Co., Wonjoo, Korea) to prevent the unabsorbed fraction being absorbed in the next segment, and the contents of the segment were removed.

**Figure 1:**
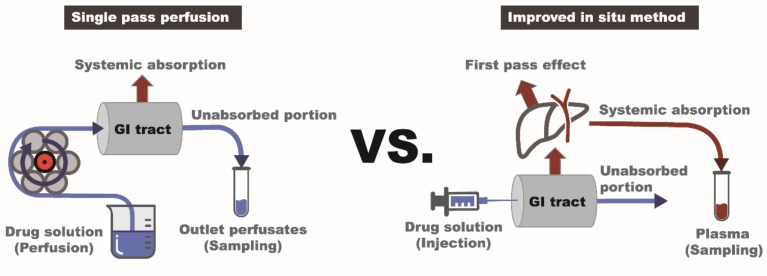
Schematic diagram of the experimental set-ups for the single-pass perfusion method and the improved in situ absorption method. While a drug solution is perfused and an outlet perfusate is sampled for the assessment of absorption in the single-pass perfusion (**left**); a drug solution is injected into the gastrointestinal segment and plasma samples are used to assess the systemic absorption of a drug in the improved in situ method (**right**).

Fimasartan dissolved in distilled water (1.0 mg/mL, 0.59 mL/kg) was injected to the starting point of the gastrointestinal segment, i.e., stomach, duodenum, small intestine, or large intestine at a dose of 0.5 mg/kg. The blood samples were collected from the jugular vein before and at 2 min, 5 min, 10 min, 15 min, 30 min, 1 h, 2 h, 4 h, and 8 h after the injection of fimasartan. Plasma samples were obtained by centrifugation of the blood samples at 1500× *g* for 10 min and were stored at −20 °C until analysis. The animal was placed on a heating pad to maintain the body temperature. The experimental set-up for the improved in situ absorption method is illustrated in comparison with that of the single-pass perfusion in Figure 1.

The relative regional fraction absorbed (*F_abs,relative_*) at each segment by the improved in situ absorption model was calculated based on the AUC values obtained following the administration of fimasartan into the segment of interest *i*:

(5) $$F_{abs,relative,\;i}=\frac{{AUC}_{i}}{{AUC}_{stomach}+{AUC}_{duodenum}+{AUC}_{small\;intestine}+{AUC}_{large\;intestine}}$$

Since the absorption occurs stepwise as drugs pass through the gastrointestinal tract from stomach to the duodenum, small intestine, and large intestine, the dose available at each segment after oral administration is reduced in the distal gastrointestinal tract. Therefore, the actual fraction absorbed in the specific segment of the gastrointestinal tract (actual *F′_abs_*) was estimated by applying the fraction of the dose arriving at the site of segment (*F_arrived_*). The *F_arrived_* at the segment of interest, *i*, was calculated as:

(6) $$F_{arrrived,\;i}=1-\sum {F\prime }_{abs,i-1}$$

*F′_abs,i−1_* is the actual fraction absorbed prior to the *ith* segment, which was estimated as:

(7) $${F\prime }_{abs,i-1}=F_{arrrived,\;i}\cdot F_{abs,relative,\;i}\cdot f$$

where *f* is a factor of 1.322, which allows the sum of *F′_abs,i_* to become the average *F_X_·F_G_*, the gastrointestinal bioavailability estimated by Equation (2).

### 2.6. LC-MS/MS

The fimasartan concentrations in rat plasma were determined by a previously validated liquid chromatography-tandem mass spectrometry (LC-MS/MS) method [6,26]. Briefly, an internal standard solution (50 μL, BR-A-563 100 ng/mL in acetonitrile) and blank acetonitrile (200 μL) was added to 50 μL of the plasma samples and mixed on a vortex mixer for 1 min. After centrifugation of the mixture for 10 min at 15,000× *g*, 100 μL of the upper layer was mixed with 100 μL of distilled water. A portion (10 μL) was injected into the LC-MS/MS.

The LC-MS/MS instrument comprised an API 4000 mass spectrometer (Applied Biosystems/MDS Sciex, Toronto, ON, Canada) coupled with an Agilent 1100 HPLC (Agilent Technologies, Santa Clara, CA, USA). Fimasartan was separated on a Kinetex C_18_ column (50 × 2.10 mm i.d., 2.6 μm, Phenomenex, Torrence, CA, USA) with a KrundKatcher ultra column inline filter (Phenomenex). The isocratic mobile phase consisted of acetonitrile and 0.05% formic acid (40:60, *v*/*v*) at a flow rate of 0.2 mL/min. The column oven temperature was set to 30 °C. The electron spray ionization (ESI) source was operated in a positive mode. The multiple reaction monitoring (MRM) transitions of precursor-to-product ion pairs were *m*/*z* 502.7→207.1 for fimasartan and *m*/*z* 526.1→207.1 for the internal standard (BR-A-563).

The LC-MS/MS method was fully validated and the lower limit of quantification was 0.2 ng/mL for rat plasma. The assay was linear over a concentration range of 0.2–500 ng/mL with correlation coefficients of >0.999. The intra- and inter-day accuracy and precision ranged from 90.8% to 108.0% and 2.4% to 13.4% for rat plasma.

### 2.7. Statistical Analysis

Data were presented as mean ± standard deviation (SD) unless otherwise stated. For comparison between the two means of the unpaired data, an unpaired *t*-test was used. Comparisons among more than two groups were performed using one-way analysis of variance (ANOVA) followed by Scheffe’s post hoc test. Statistical significance was denoted when *p* < 0.05.

## 3. Results

### 3.1. Determination of Gastrointestinal Bioavailability (F_X_·F_G_)

**Figure 2:**
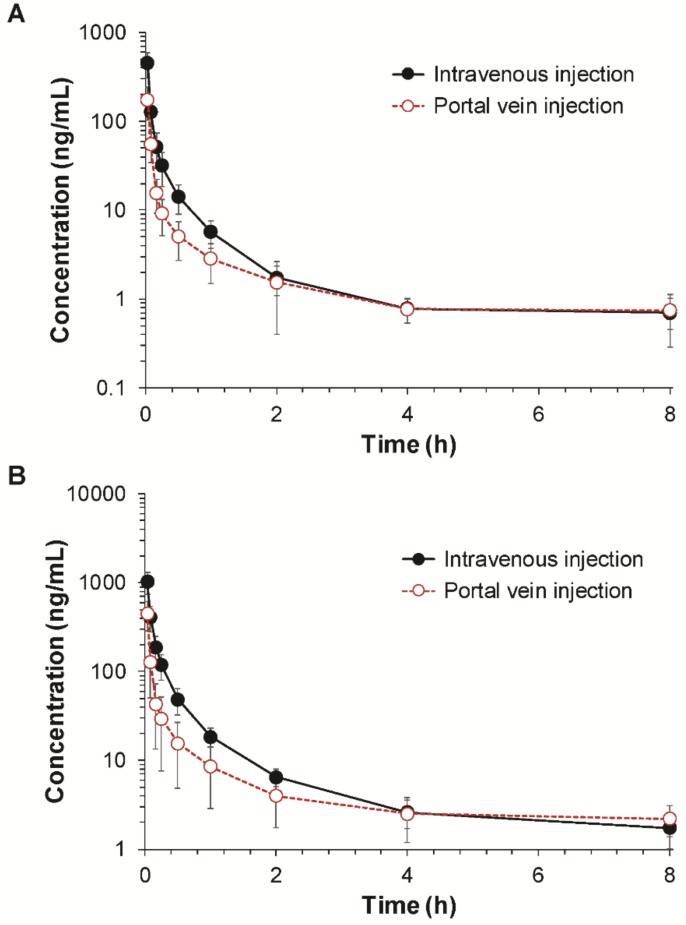
Plasma concentration–time profiles of fimasartan following the intravenous and portal venous injections of fimasartan at doses of (**A**) 0.1 mg/kg (*n* = 8–9) and (**B**) 0.3 mg/kg (*n* = 5–8) in rats (mean ± SD).

The average plasma concentration–time profiles of fimasartan obtained following the intravenous and portal vein injections of fimasartan are depicted in Figure 2. The noncompartmental pharmacokinetic parameters of fimasartan are summarized in Table 1. Following the intravenous injection, plasma concentrations of fimasartan showed a multiexponential decline with the mean elimination half-life (t_1/2_) of 3.12–3.88 h. The initial concentration (C_0_) and the AUC values increased with the dose increase. The plasma concentration–time profiles of fimasartan after the portal vein injection declined with the mean t_1/2_ of 4.20–4.61 h, which is comparable with that observed after the intravenous injection. However, the C_0_ and AUC obtained following portal vein injection were significantly lower than that obtained following intravenous injection, which indicated that a significant amount of fimasartan underwent hepatic first-pass metabolism.

Based on the AUC values after the intravenous and portal vein injections, the fractions of the administered dose that escaped the first-pass metabolism in the liver (*F_H_*) were calculated as 46.63% and 48.13% at doses of 0.1 and 0.3 mg/kg, respectively. The mean *F_H_* was estimated at 47.35% (Table 1). Then, the gastrointestinal bioavailability (*F_X_·F_G_*) was estimated at 84.1% based on the absolute oral bioavailability of fimasartan, which is 39.85% in rats [6], by using Equation (2).

### 3.2. The Gastrointestinal Permeability of Fimasartan Determined by Using the Single-Pass Perfusion

**Figure 3:**
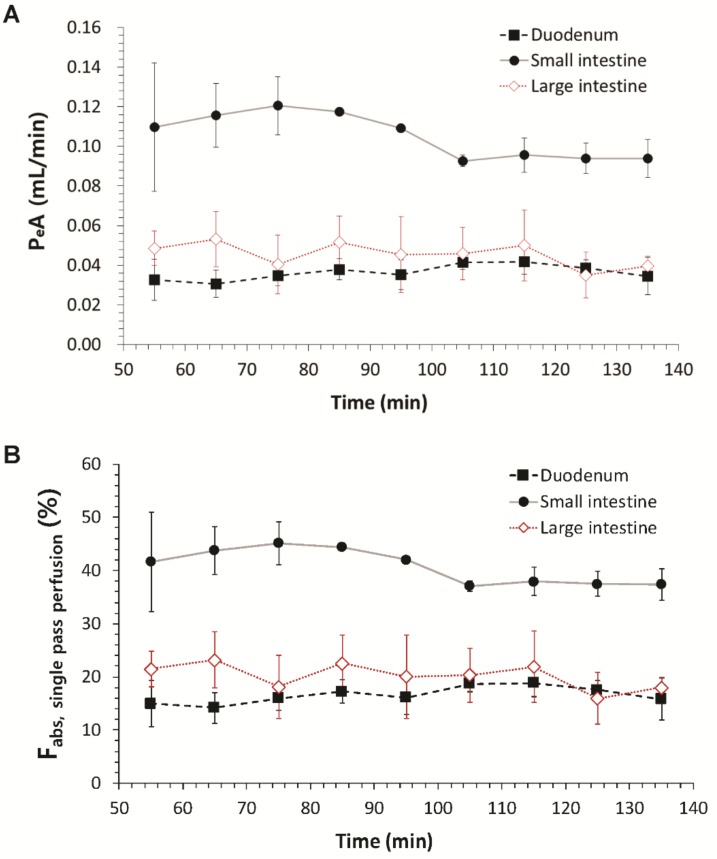
Regional absorption clearance (*P_e_A*) and the fraction absorbed (*F_abs_*) in the duodenum, small intestine, and large intestine in rats by the in situ single-pass perfusion (*n* = 4, mean ± SD). *P_e_A* was calculated by *Q*·ln(*C_in_*/*C_out_*), where *Q* is the perfusion rate, and *C_in_* and *C_out_* are the inlet and outlet concentrations, respectively. *F_abs_* was calculated by (*C_in_*–*C_out_*)/*C_in_*.

The regional absorptions of fimasartan in the duodenum, small intestine, and large intestine were evaluated by in situ single-pass intestinal perfusion. The absorption clearance (*P_e_A*) and the fraction absorbed (*F_abs_*) of fimasartan through the duodenum, small intestine, or large intestine after initiation of the perfusion are shown in Figure 3. The outflow drug concentration reached the steady state within 1 h after the initiation of the perfusion. The steady-state *P_e_A* and *F_abs_* were calculated based on *C_in_* and *C_out_* at 90 min. The steady-state *P_e_A* and *F_abs_* values of fimasartan in the different gastrointestinal regions determined by the in situ single-pass perfusion method are shown in Table 2.

The highest *P_e_A* value was observed in the small intestine followed by large intestine and duodenum. The *P_e_A* value in the small intestine was 2.71- and 2.36-fold higher than those in the duodenum and large intestine, respectively. The *F_abs_* was also the highest in the small intestine, indicating 37.38% of the administered dose into the small intestine was absorbed, which was 2.37- and 2.08-fold higher compared with those in the duodenum and large intestine, respectively (Table 2).

### 3.3. Regional Absorption Fraction of Fimasartan Determined by Using the Improved In Situ Absorption Model

**Figure 4:**
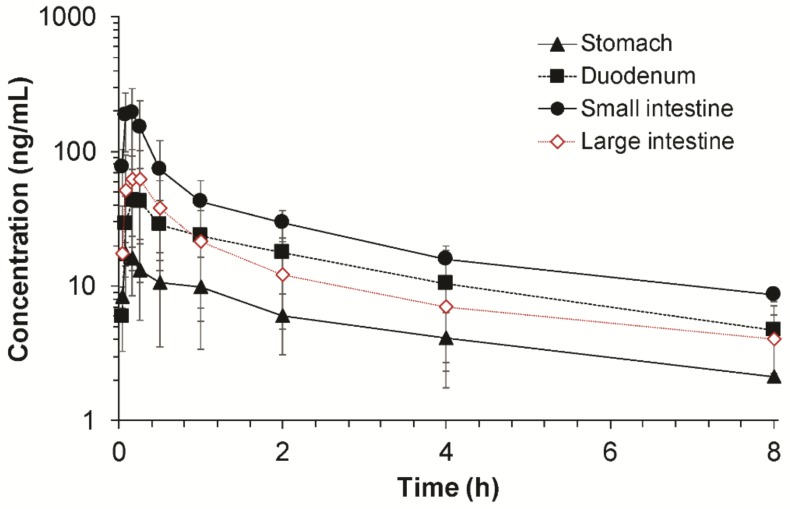
Plasma concentration–time profiles of fimasartan following administration of fimasartan (0.5 mg/kg) into each gastrointestinal segment in rats by the improved in situ absorption model (*n* = 4–6, mean ± SD).

Plasma concentration–time profiles of fimasartan following the administration of fimasartan (0.5 mg/kg) into the different gastrointestinal segments, i.e., stomach, duodenum, small intestine, and large intestine, are depicted in Figure 4. The corresponding noncompartmental pharmacokinetic parameters of fimasartan are summarized in Table 3.

Following administration of fimasartan into the specific gastrointestinal segment, the fimasartan concentration in the plasma rapidly increased, reached the peak concentration within 20 min, and declined after that, regardless of the administration segment (Figure 4). The decline of the fimasartan concentrations in the plasma also appeared to be parallel among the administration segment. The estimated t_1/2_ of fimasartan was ranged from 3.11 ± 0.88 h to 4.30 ± 1.61 h, which were comparable with the t_1/2_ obtained after the intravenous injection (Table 1). On the other hand, the maximum concentration (C_max_) and AUC values of fimasartan were observed to be significantly different among different gastrointestinal segments, in which fimasartan was administered. The administration of fimasartan into the small intestine resulted in the highest overall plasma concentration (*p* < 0.05). The highest C_max_ was observed after the fimasartan administration into the small intestine followed by the large intestine, duodenum, and stomach. The C_max_ obtained after the small intestine administration was 2.84-, 4.29-, and 10.45-fold higher than those obtained after the large intestine, duodenum, and stomach administration, respectively. Similarly, the administration of fimasartan into the small intestine segment also resulted in the greatest AUC values while the stomach administration resulted in the smallest AUC values. The AUC_inf_ obtained after the small intestine administration was 2.60-, 2.16-, and 6.03-fold higher than those obtained after the large intestine, duodenum, and stomach administration, respectively (Table 3).

The relative fraction absorbed (*F_abs,relative_*) of fimasartan in the specific gastrointestinal region was estimated based on the AUC values obtained after administration into the corresponding gastrointestinal segment compared to the sum of AUC values (Equation (5)). The calculated *F_abs,relative_* of fimasartan in different gastrointestinal regions are shown in Table 4. The highest *F_abs,relative_* of 49.7% ± 11.5% was obtained in the small intestine, indicating that the small intestine was responsible for approximately 49.7% ± 11.5% of the fimasartan absorption in the gastrointestinal tract followed by the duodenum (23.0% ± 12.1%), large intestine (19.1% ± 11.9%), and stomach (8.2% ± 3.2%).

The actual fraction absorbed (actual *F′_abs_*) in a specific gastrointestinal region accounting for the reduced amount of dose arriving in the gastrointestinal segment (*F_arrived_*) due to absorption at the previous segment is summarized in Table 4. The sum of actual *F′_abs_* was set to be the estimated gastrointestinal bioavailability (*F_X_·F_G_*) of 84.1% (Table 1) and the factor (f) was 1.322. As shown in Table 4, the actual *F′_abs_* values were estimated as 10.9%, 27.1%, 40.7%, and 5.4% in the stomach, duodenum, small intestine, and large intestine, respectively. The majority of the fimasartan dose (67.8%) was predicted to be absorbed in the duodenum and small intestine. The results indicated that 10.9% of the orally administered fimasartan was absorbed in the stomach and the remaining 89.1% arrived at the duodenum where 27.1% was absorbed. Then, 40.7% and 5.4% of the dose were absorbed in the small intestine and large intestine, respectively (Table 4).

## 4. Discussion

The regional absorption of fimasartan in the gastrointestinal tract was evaluated by an improved in situ absorption method in rats. The results were also compared with those determined by a conventional in situ single-pass perfusion method. The improved in situ approach measured the drug concentration in the plasma following the injection of fimasartan into a specific part of the gastrointestinal tract (Figure 1) and provided an accurate assessment of the absorbed fraction of a drug into the systemic circulation across the region of gastrointestinal tract after oral administration.

Before estimating the regional absorption, the gastrointestinal bioavailability (*F_X_·F_G_*) was determined first based on the ratio of the AUC values following the portal vein and intravenous injections. The *F_X_·F_G_* consists of the fraction absorbed (*F_X_*) and the fraction that is not metabolized during passage through the gut wall (*F_G_*) [1]. The estimated gastrointestinal bioavailability (*F_X_·F_G_*) of fimasartan was 84.1% in rats, whereas over 50% of the dose was eliminated by the first-pass metabolism in the liver (Table 1). These results are in agreement with the previous studies, which indicated the extensive fecal excretion of fimasartan because of the biliary excretion rather than a low gastrointestinal absorption and an extensive absorption of orally administered fimasartan in the gastrointestinal tract [6].

To estimate the regional absorption with the improved in situ absorption method, the relative fraction absorbed in each gastrointestinal segment (*F_abs,relative_*) was determined by using the AUC obtained after injection of fimasartan into the segment. Since absorption occurs sequentially along the gastrointestinal tract, the actual fraction absorbed (*F′_abs_*) was finally estimated by applying the fraction of dose available in each segment (*F_arrived_*). Our results indicated that the orally administered fimasartan was absorbed in the stomach (10.9%), duodenum (27.1%), small intestine (40.7%), and large intestine (5.4%) as the drug passed through the gastrointestinal tract, which added up to the gastrointestinal availability (*F_X_·F_G_*) of 84.1% (Table 4). Although the *F_abs,relative_* indicated that the absorption potential of fimasartan of the large intestine was comparable with that of the duodenum, the actual fraction absorbed in the large intestine (*F′_abs_*) was much less than that in the duodenum. The *F′_abs_* in the large intestine was smaller because only 21.3% of the orally administered drug was available in the large intestine due to the absorption in the stomach, duodenum, and small intestine before the drug entered the large intestine. Taken together, in case of immediate release formulation, the majority of the orally administered fimasartan (67.8%) was predicted to be absorbed in the duodenum and small intestine. However, the comparable absorption potential of the large intestine (*F_abs,relative,LI_* = 19.1% ± 11.9%) as the upper part of the gastrointestinal tract (*F_abs,relative,duodenum_* = 23.0% ± 12.1%) suggested that sufficient absorption may be expected in the lower part of the gastrointestinal tract in case of extended release formulation. For the development of extended release formulations, sufficiently high absorption of the drug in both upper and lower parts of the gastrointestinal tract is needed to achieve the desired therapeutic effects [21].

**Figure 5:**
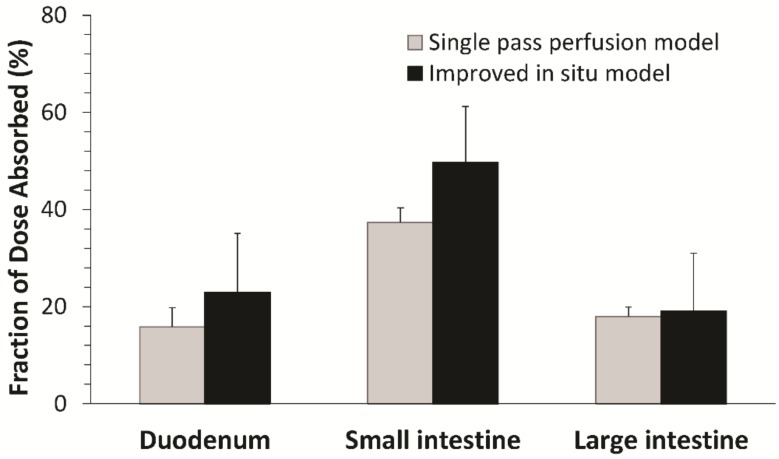
Comparison between the relative fraction absorbed (*F_abs,relative_*) by the improved in situ absorption model and the fraction absorbed (*F_abs_*) determined by the single-pass perfusion (*n* = 4–6, mean ± SD).

The regional absorption of fimasartan determined by the improved in situ absorption model was in good agreement with that by the conventional in situ single pass perfusion technique (Figure 5). In both methods, the highest absorption was predicted through the small intestine while the absorptions in the duodenum and large intestine were similar. There were no significant differences between the regional absorptions of fimasartan determined by the two methods in each gastrointestinal segment. The results of the two methods were comparable, because the model drug in the present study, fimasartan, may be minimally metabolized in the gastrointestinal tract. However, for drugs that undergo extensive first-pass metabolism in the gut wall, the results may be different. As the single-pass perfusion determined the disappearance of the drug in the perfusate, which is a net result of the absorption and metabolic degradation as an indicator of drug absorption, it may overestimate the absorption. On the contrary, the present improved in situ absorption model directly determined the resulting plasma concentrations considering both intestinal permeability and metabolism, leading to more accurate estimations of gastrointestinal bioavailability and regional absorption.

Another advantage of the present improved in situ absorption method is that the drug was injected into the specific part of the gastrointestinal lumen instead of perfusion, which represents a more physiological absorption process. During perfusion, the gastrointestinal lumen is filled with the perfusate, which may disturb the normal physiology of the gastrointestinal tract. Moreover, since the lumen is filled with the drug solution, the drug absorption presumably occurs simultaneously in the whole gastrointestinal tract. However, the real drug absorption in the gastrointestinal tract in vivo is a sequential process along the gastrointestinal tract. In the improved in situ absorption method, therefore, by injecting the drug solution into the starting point of the segment, gradually less amount of drug would be applied to the gastrointestinal tract as the drug solution passes through the gastrointestinal tract after injection, which is close to the real drug absorption condition without disturbing gastrointestinal physiology.

In addition, the improved in situ absorption model has advantages compared to the single-pass perfusion method in terms of the amount of the test drug compound needed. In the improved in situ model, the drug is administered by a single injection into the region of interest, while the single pass perfusion method needs perfusion of a drug until the steady state is reached. Thus, the improved in situ absorption model requires less amount of drug than the single-pass perfusion. The characteristics of the improved in situ absorption method are summarized in comparison with the single-pass perfusion method in Table 5.

A better understanding of the regional absorption of a drug provides useful insight for the formulation development. The importance of good regional absorption characteristics, which is high and similar absorption throughout the gastrointestinal tract, of a selected compound may be crucial for the development of extended release formulations [27]. If the absorption is limited in the certain part of the gastrointestinal tract, formulations that make a drug stay for longer time at the absorption site may be designed to increase the absorption time, thereby improving the bioavailability. For example, drugs that are efficiently absorbed in the upper gastrointestinal tract may be formulated as gastroretentive systems to improve oral bioavailability, where extended release formulations may not help.

In summary, a novel improved in situ absorption method was developed for the assessment of regional absorption in the gastrointestinal tract by using fimasartan as a model drug. Instead of measuring drug concentrations in the perfusate while a segment of the intestine is perfused with a drug solution, this method measured the actual plasma drug concentration following the injection of fimasartan into a specific part of the gastrointestinal tract. Thus, the present approach provides more physiological and accurate assessment of the absorbed fraction into the systemic circulation across the region of the gastrointestinal tract after the oral administration. The developed improved in situ absorption model would provide a useful experimental strategy to understand the regional absorption of a drug and a guide to developing new formulations with optimized oral bioavailability.

## Figures and Tables

**Table 1:** Noncompartmental pharmacokinetic parameters of fimasartan obtained after the intravenous (I.V.) and portal vein (P.V.) injections of fimasartan in rats (mean ± SD).

Parameter	0.1 mg/kg		0.3 mg/kg	
	I.V. (*n* = 9)	P.V. (*n* = 8)	I.V. (*n* = 8)	P.V. (*n* = 5)
t_1/2_ (h)	3.88 ± 1.61	4.61 ± 2.05	3.12 ± 0.38	4.20 ± 1.61
C_0_ (ng/mL)	1074.14 ± 339.75	385.35 ± 184.74 *	1922.84 ± 573.3	1068.57 ± 197.78 *
AUC_all_ (ng·h/mL)	70.68 ± 19.96	30.26 ± 10.41 *	190.89 ± 51.04	82.55 ± 37.03 *
AUC_inf_ (ng·h/mL)	74.96 ± 22.37	34.95 ± 11.89 *	198.92 ± 54.22	95.74 ± 41.52 *
CL_s_ (mL/min/kg)	24.94 ± 10.89	57.17 ± 33.87 *	26.44 ± 5.7	61.84 ± 28.5 *
V_ss_ (L/kg)	1.73 ± 1.1	10.39 ± 8.03 *	1.67 ± 0.48	9.71 ± 6.79 *
*F_H_* (%)		46.63		47.38
*F_X_·F_G_* (%)		85.46		82.79

* *p* < 0.05 vs. I.V. injection.

**Table 2:** Absorption clearance (*P_e_A*) and fraction absorbed (*F_abs_*) of fimasartan in different gastrointestinal regions determined by single-pass perfusion (*n* = 4, mean ± SD).

	Single Pass Perfusion Model	
Absorption Site	*P_e_A* (mL/min)	*F_abs_* (%)
	$P_{e}A=Q_{in}\cdot ln(\frac{C_{in}}{C_{out}})$	$F_{abs,\;i}=\frac{C_{in}-C_{out}}{C_{in}}$
Duodenum	0.0346 ± 0.0095	15.80 ± 3.95
Small intestine	0.0938 ± 0.0096	37.38 ± 3.00
Large intestine	0.0397 ± 0.0048	17.98 ± 1.95

*P_e_A*, absorption clearance at 140 min after the initiation of the perfusion; *F_abs,i_*, fraction absorbed in the gastrointestinal segment of interest.

**Table 3:** Noncompartmental pharmacokinetic parameters of fimasartan obtained after administration of fimasartan (0.5 mg/kg) into specific gastrointestinal segments in rats (mean ± SD).

Parameter	Stomach (*n* = 4)	Duodenum (*n* = 4)	Small Intestine (*n* = 4)	Large Intestine (*n* = 6)
t_1/2_ (h)	3.42 ± 2.05	3.53 ± 1.54	4.30 ± 1.61	3.11 ± 0.88
T_max_ (h)	0.13 ± 0.05	0.29 ± 0.14 *	0.13 ± 0.05	0.18 ± 0.06
C_max_ (ng/mL)	18.58 ± 4.87	45.25 ± 30.75	194.25 ± 98.59 **	68.37 ± 41.26
AUC_all_ (ng·h/mL)	31.64 ± 13.75	108.31 ± 71.83	211.45 ± 76.44 **	95.14 ± 63.69
AUC_inf_ (ng·h/mL)	48.43 ± 19.10	135.22 ± 71.04	292.15 ± 67.81 **	112.33 ± 69.84

* *p* < 0.05 vs. stomach and small intestine; ** *p* < 0.05 vs. stomach, duodenum and large intestine.

**Table 4:** Fraction absorbed (*F_abs_*) of fimasartan in different gastrointestinal regions determined by the improved in situ absorption model (*n* = 4, mean ± SD).

	Relative *F_abs,relative_*	*F_arrived_*	Actual *F′_abs_*
Absorption Site	$F_{arrrived,\;i}=\frac{{AUC}_{i}}{\sum {AUC}_{i}}$	$F_{arrrived,\;i}=1-\sum {F\prime }_{abs,\;i-1}$	${F\prime }_{abs,i}=F_{arrrived,\;i}\cdot F_{abs,relative,\;i}\cdot f$
Stomach	*F_abs,relative,sto_* = 8.2 ± 3.2%	100%	*F′_sto_* = 1·*F_abs,relative,sto_·ƒ* = 10.9%
Duodenum	*F_abs,relative,duo_* = 23.0 ± 12.1%	1 − *F′_abs,sto_* = 89.1%	*F′_duo_* = *F_arrived,duo_·F_abs,relative,duo_·ƒ* = 27.1%
Small intestine	*F_abs,relative,SI_* = 49.7 ± 11.5%	1 − (*F′_abs,sto_* + *F′_abs,duo_*) = 62.0%	*F′_SI_* = *F_arrived,SI_·F_abs,relative,SI_v·ƒ* = 40.7%
Large intestine	*F_abs,relative,LI_* = 19.1 ± 11.9%	1 − (*F′_abs,sto_* + *F′_abs,duo_* + *F′_abs,SI_*) = 21.3%	*F′_LI_* = *F_arrived,LI_·F_abs,relative,LI_·ƒ* = 5.4%
Sum	100.0%	-	*F_X_·F_G_* = 84.1%

*F_abs,relative_*, relative fraction absorbed in the gastrointestinal segment of interest; *F_arrived_*, fraction arriving at the gastrointestinal segment of interest; *F′_abs_*, actual fraction absorbed in the gastrointestinal segment of interest corrected by the fraction arriving; *f*, factor = 1.332.

**Table 5:** Comparison of the improved in situ absorption method and the single-pass perfusion method for evaluation of the regional absorption.

	Single-Pass Perfusion Method	Improved In Situ Absorption Method
Administration of a drug	A segment of the gastrointestinal tract is perfused with a drug solution. Normal physiology of the gastrointestinal tract may be disturbed during perfusion. After filling the gastrointestinal lumen with the perfusate, drug absorption occurs simultaneously in the whole gastrointestinal tract.	A drug solution is injected into a segment of the gastrointestinal tract. Normal physiology of the gastrointestinal tract would maintain. Drug absorption occurs sequentially as the drug solution passes through the gastrointestinal tract.
Estimation of the absorption	The absorption is determined by the difference between the drug concentration in the perfusate entering and that leaving the segment Drug metabolism and degradation are neglected. The absorption may be overestimated for drugs that undergo significant gastrointestinal metabolism and degradation.	The absorption is directly determined by the area under the plasma drug concentrations vs. time curves. Drug metabolism and degradation affecting plasma drug concentration are comprehensively considered. More accurate gastrointestinal bioavailability and regional absorption could be estimated.
Amount of the test drug needed	A significant amount of drug should be perfused until reaching the steady state.	Less amount of the drug is required.
